# Synthesis and Evaluation of the Tumor Cell Growth Inhibitory Potential of New Putative HSP90 Inhibitors

**DOI:** 10.3390/molecules23020407

**Published:** 2018-02-13

**Authors:** Ana Bizarro, Diana Sousa, Raquel T. Lima, Loana Musso, Raffaella Cincinelli, Vantina Zuco, Michelandrea De Cesare, Sabrina Dallavalle, M. Helena Vasconcelos

**Affiliations:** 1 Department of Biological Sciences, Faculty of Pharmacy of the University of Porto (FFUP), Rua de Jorge Viterbo Ferreira 228, 4050-313 Porto, Portugal; sofiabizarro@gmail.com (A.B.); dsousa@ipatimup.pt (D.S.); 2 Department of Biology, School of Sciences, University of Minho, 4710-057 Braga, Portugal; 3 i3S—Instituto de Investigação e Inovação em Saúde da Universidade do Porto, Rua Alfredo Allen 208, 4200-135 Porto, Portugal; rlima@ipatimup.pt; 4 Cancer Drug Resistance Group, Institute of Molecular Pathology and Immunology of the University of Porto (IPATIMUP), 4200-135 Porto, Portugal; 5 Department of Pathology, Faculty of Medicine of the University of Porto (FMUP), Alameda Prof. Hernâni Monteiro, 4200-319 Porto, Portugal; 6 Department of Food, Environmental and Nutritional Sciences Division of Chemistry and Molecular Biology, Università degli Studi di Milano, via Celoria 2, 20133 Milano, Italy; loana.musso@unimi.it (L.M.); raffaella.cincinelli@unimi.it (R.C.); 7 Department of Experimental Oncology and Molecular Medicine, Fondazione, IRCCS—Istituto Nazionale dei Tumori, Via Amadeo 42, 20133 Milano, Italy; valentina.zuco@istitutotumori.mi.it (V.Z.); Andrea.DeCesare@istitutotumori.mi.it (M.D.C.)

**Keywords:** HSP90, naphthoquinones, (thio)chromenopyridinones, antitumor, cell proliferation, cell cycle, apoptosis, tumor xenographs

## Abstract

Background: Heat shock protein 90 (HSP90) is a well-known target for cancer therapy. In a previous work, some of us have reported a series of 3-aryl-naphtho[2,3-*d*]isoxazole-4,9-diones as inhibitors of HSP90. Methods: In the present work, various compounds with new chromenopyridinone and thiochromenopyridinone scaffolds were synthesized as potential HSP90 inhibitors. Their binding affinity to HSP90 was studied in vitro. Selected compounds (**5** and **8**) were further studied in various tumor cell lines regarding their potential to cause cell growth inhibition, alter the cell cycle profile, inhibit proliferation, and induce apoptosis. Their effect on HSP90 client protein levels was also confirmed in two cell lines. Finally, the antitumor activity of compound **8** was studied in A431 squamous cell carcinoma xenografts in nude mice. Results: Our results indicated that treatment with compounds **5** and **8** decreased the proliferation of tumor cell lines and compound **8** induced apoptosis. In addition, these two compounds were able to downregulate selected proteins known as “clients” of HSP90. Finally, treatment of xenografted mice with compound **5** resulted in a considerable dose-dependent inhibition of tumor growth. Conclusions: Our results show that two new compounds with a chromenopyridinone and thiochromenopyridinone scaffold are promising putative HSP90 inhibitors causing tumor cell growth inhibition.

## 1. Introduction

Heat shock proteins (HSPs) are key elements in protein synthesis, acting in protein folding and in the stabilization of newly synthesized proteins [1]. The expression of HSPs is induced as a response to environmental stress conditions [2] and they have been associated with tumor cell proliferation, differentiation, metastatic spread, invasion, and drug resistance [3,4]. Since tumor cells must extensively renew their metabolic and signal transduction pathways, they are more dependent on “stress-related proteins” (such as HSPs) than non-tumor cells [4]. Indeed, overexpression of HSPs was described in several types of cancer [5]. 

Amongst the heat-shock protein family, the heat-shock protein 90 (HSP90) is the most widely studied target for cancer therapy, being important to the maturation and stabilization of different client oncoproteins such as Akt, Bcr-Abl, HER-2/ErbB2, Raf-1, CDK, survivin, and mutated p53 [6]. HSP90 overexpression was related to medulloblastoma, breast cancer and colorectal cancer [3]. Thus, HSP90 has attracted a great deal of interest as a potential anticancer target. The HSP90 chaperone cycle is a dynamic process mediated by interactions with co-chaperones and other binding partners to form a multi-chaperone complex machinery. HSP90 exists in diverse conformational states and co-chaperones may function by favoring distinct conformations that help to drive the cycle [7]. The dynamic nature of the cycle allows modulation by small molecule ligands through a number of distinct mechanisms that include targeting the *N*-terminal domain, the C-terminal domain, co-chaperone binding and client protein binding. HSP90 inhibitors based on the benzoquinone ansamycin scaffold of geldanamycin, the resorcinol scaffold of radicicol, the purine scaffold of ATP, and other unique chemical scaffolds which were discovered to inhibit HSP90, are currently being evaluated in numerous ongoing or completed clinical trials [8,9]. While some of the results from such trials have been encouraging, reports of hepato-, cardio-, ocular-toxicity, peripheral neuropathy and in some cases poor solubility, limited bioavailability and difficult dosing schedules have discontinued clinical development, and to date, no HSP90 inhibitor has obtained regulatory approval [7].

Therefore, due to HSP90’s role in maintaining the viability of cancer cells, the search for novel and more efficient HSP90 inhibitors represents an interesting challenge from a drug discovery perspective.

Quinones are a class of organic compounds endowed with a variety of biological activities, mostly connected with their redox properties. A number of natural and synthetic quinones showed remarkable anticancer activity, and 1,4-naphthoquinones have been identified as HSP90 inhibitors [10,11,12]. In a previous work, some of us have reported a series of 3-aryl-naphtho[2,3-*d*]isoxazole-4,9-diones as inhibitors of HSP90 [13]. As a continuation of our previous investigation, in the present work a series of new pyridine-fused naphthoquinones, chromenopyridinones and thiochromenopyridinones, were synthesized aiming to enhance their ability to inhibit HSP90 and to reduce tumor cell growth both in vitro and in vivo.

## 2. Results and Discussion

### 2.1. Chemistry

Initially, we had planned to replace the isoxazole group fused to the 1,4-naphthoquinone ring [3] in previously reported isoxazolonaphtoquinones with a pyridine moiety (**1**)). The advantage of the replacement could be the possibility of *N*-alkylation to give the corresponding ammonium salts, possibly more water soluble.

**Scheme 1:**
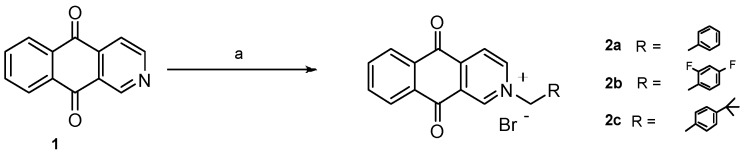
Synthesis of *N*-substituted benzo[g]isoquinoline-5,10-diones*. Reagents and conditions*: (a) for **2a**: benzyl bromide, DMSO, r.t., 48 h, 86%; for **2b**: 2-bromomethyl-1,4-difluorobenzene, DMSO, r.t., 24 h, 60%; for **2c**: 1-bromomethyl-4-*tert*-butylbenzene, DMSO, 24 h, r.t., 65%.

Thus, we prepared compounds **2a**–**c** by treatment of commercially available benzo[*g*]isoquinoline-5,10-dione (**1**) with benzyl bromides in DMSO at room temperature (Scheme 1).

**Scheme 2:**
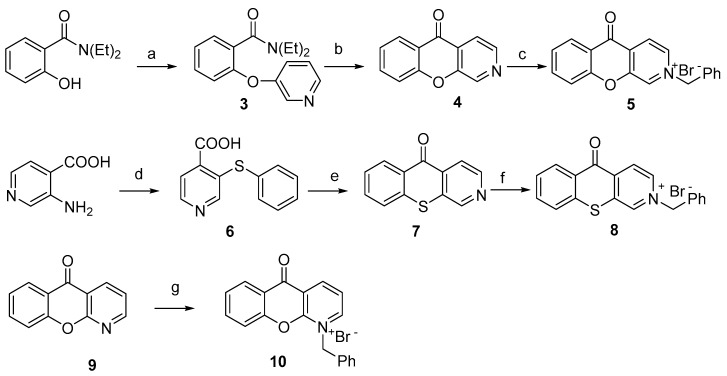
Synthesis of (thio)chromenopyridinones. *Reagents and conditions*: (a) 3-bromopyridine, KO*t*Bu, 210 °C, 3 h, 74%; (b) LDA, THF, 0 °C, then 1 h at r.t., 90%; (c) benzyl bromide, DMSO, r.t., 48 h, 86%; (d) i: NaNO_2_, H_2_O, HCl conc., 5 °C; ii: thiophenol, NaOH, H_2_O, reflux, 3 h; (e) i: SOCl_2_, 1 h, reflux; ii: AlCl_3_, PhNO_2_, 100 °C, 4 h, 72%; (f) benzyl bromide, DMSO, overnight at r.t., 55%; (g) benzyl bromide, DMSO, overnight at r.t., 34%.

Successively, we planned to investigate the role of the quinone nucleus by replacing it with a heterocyclic ring. Changing the quinone structure into a xanthone or thioxanthone could reduce the risk of idiosyncratic drug reactions [14]. Compound **4** was obtained starting from *N*,*N*-diethyl-2-hydroxybenzamide, which was converted into compound amide **3** by reaction with 3-bromopyridine in the presence of KO*t*Bu at 210 °C. An LDA-induced regiospecific acylation of **3** gave the corresponding 5*H*-chromeno[2,3-*c*]pyridin-5-one **4**. Alkylation with benzyl bromide afforded chromenopyridinone **5**. To prepare thiochromenopyridinone **7** diazotised 3-aminoisonicotinic acid was condensed with thiophenol in alkaline solution to form 3-(phenylthio)isonicotinic acid **6**. This was converted into the corresponding acyl chloride by treatment with thionyl chloride. Heating in nitrobenzene with AlCl_3_ afforded compound **7**, which was then alkylated with benzyk bromide to obtain **8** in 55% yield (Scheme 2).

### 2.2. Binding Affinity to HSP90, Cell Growth Inhibitory Activity and Structure–Activity Relationship Studies of the Newly Synthesized Compounds

The prepared compounds were tested for their affinity to HSP90 (with a binding assay) and for their cell growth inhibitory activity (with the cell counting assay) on a number of human tumor cell lines (non-small cell lung cancer NCI-H460, squamous-cell carcinoma A431 and peritoneal mesothelioma STO). Results are presented in Table 1.

Unfortunately, for some of the compounds (e.g., compounds **2a**–**c**) the binding affinity assay did not give valuable results since the compounds themselves were autofluorescent. However, compounds **2a**–**c** showed significant cell growth inhibitory activity. As these results suggested that other off-target effects are likely involved in the mechanism of action of the tested compounds, we planned to investigate the role of the quinone nucleus by replacing it with a xanthone moiety (compound **4**). This replacement led to a complete loss of cell growth inhibitory activity and binding affinity to HSP90. However, the introduction of the benzyl group (compound **5**) restored the cell growth inhibitory activity, in spite of lack of evidence of HSP90 binding. The position of the nitrogen was crucial; in fact, shifting the nitrogen atom from position 2 (compound **5**) to 1 (compound **10**) completely abolished the activity. 

Interestingly, compound **7**, with a sulphur atom in place of the oxygen in the xanthone ring showed a high affinity to HSP90, in spite of the low cell growth inhibitory activity. In this case, the chemico-physical features of the compound may influence the cellular pharmacokinetic behavior and therefore cellular response. Therefore, the corresponding alkylated compound **8** was synthesized. This compound showed appreciable cell growth inhibition associated with a strong HSP90 interaction.

Compounds **5** and **8** appeared to be some of the most promising ones regarding tumor cell growth inhibition, thus we further investigated their cellular/biochemical profile of activity.

### 2.3. Compounds ***5*** and ***8*** Induced Cytotoxicity in Different Tumor Cell Lines

Cytotoxic effects of the compounds against MCF7 (breast adenocarcinoma), AGS (gastric adenocarcinoma) and NCI-H460 (non-small cell lung cancer) cells were evaluated after 48 h of treatment, using the SRB assay. Cytotoxicity of each compound was evaluated by its GI_50_ (concentration which inhibits cell growth by 50%). The compounds presented GI_50_ bellow 7 μM in all the cell lines tested (Table 2).

Moreover, in order to verify the possible toxicity of the compounds for normal cells, their growth inhibitory effect was also analyzed in the non-tumor human fibroblast cell line HFF-1. Both compounds presented no cytotoxicity towards this cell line after seven days of treatment (data not shown).

**Figure 1:**
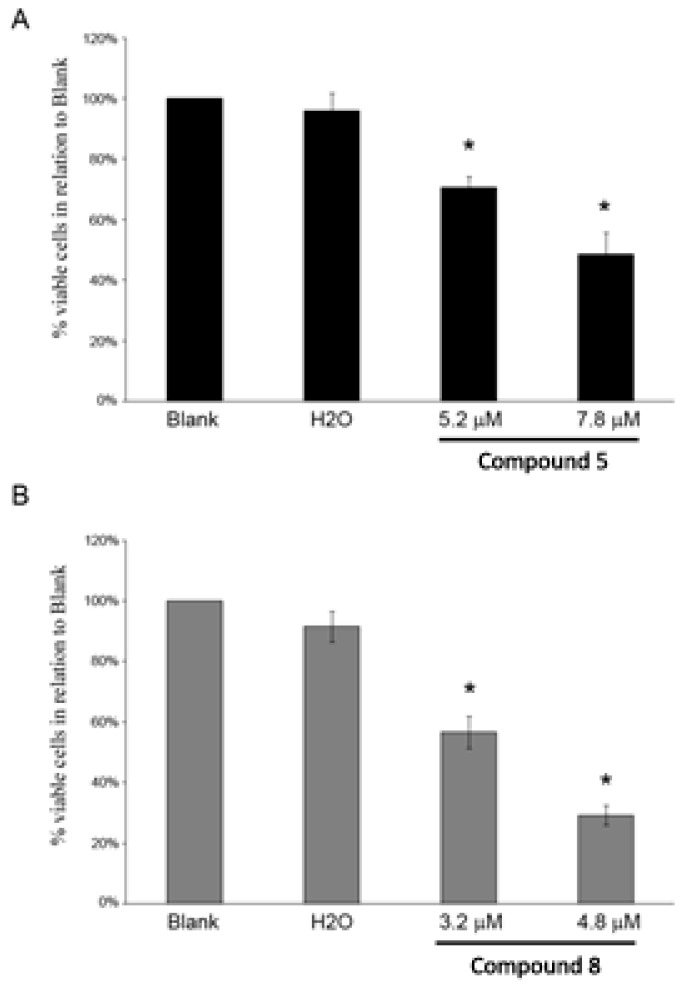
NCI-H460 viable cell number 48 h following treatment with compounds **5** (**A**) and **8** (**B**) analyzed with trypan blue exclusion assay. Cells were treated with the GI_50_ (5.2 μM) and 1.5× GI_50_ (7.8 μM) of compound **5** and with the GI_50_ (3.2 μM) and 1.5× GI_50_ (4.8 μM) of compound **8**. Cells were also treated with the corresponding highest concentration of vehicle (solvent) of the compounds (H_2_O). Results are presented as % of viable cells in relation to blank cells (treated with medium) and are the mean ± SEM of six independent experiments. * *p* ≤ 0.001 Blank vs. treatment.

The cell growth inhibitory effect of compounds **5** and **8** was further investigated in the NCI-H460 cell line. This study confirmed that both these compounds decreased NCI-H460 viable cell number, as previously indirectly indicated by the SRB assay. By counting the number of viable cells with trypan blue 48 h following treatment, it was possible to observe that compound **5** at 5.2 μM and 7.8 μM (corresponding to the previously determined GI_50_ and to 1.5× GI_50_ concentration, respectively) significantly reduced the number of viable cells to 71% and 49% (in relation to Blank cells), respectively (Figure 1A). Likewise, this decrease in the number of viable cells was also observed following treatment with compound **8** at 3.2 μM and 4.8 μM (corresponding to the previously determined GI_50_ and to 1.5× GI_50_ concentration, respectively) to 57% and 29% (in relation to Blank cells), respectively (Figure 1B).

### 2.4. Apoptotic Induction in NCI-H460 Cells after Treatment with Compound ***8***

Anticancer drugs exert at least part of their cytotoxic effect by triggering apoptosis. To assess if the growth inhibition observed after compounds treatment was due to apoptosis, cells were treated with two concentrations of compounds **5** and **8** (corresponding to GI_50_ and 1.5× GI_50_) for 48 h and apoptosis was detected with Annexin V and propidium iodide double staining by flow cytometry. Annexin (+) and PI (+) cells were not found in compound **5** treated cells (Table 3(A)), suggesting that this compound did not induce apoptosis at cytotoxic concentrations. However, Annexin (+) and PI (+) cells were detected in compound **8** treated cells, indicating that this compound induced apoptosis at cytotoxic concentrations (Table 3(B)).

The reason why compound **8** is a stronger inducer of apoptosis than compound **5** might be related to differences in the affinity for HSP90. This cannot be confirmed since it was not possible to determine the capacity of compound **5** to bind HSP90 due to its autofluorescence (Table 1).

### 2.5. Effect of the Compounds ***5*** and ***8*** on NCI-H460 Cell Cycle Profile and Cellular Proliferation

**Figure 2:**
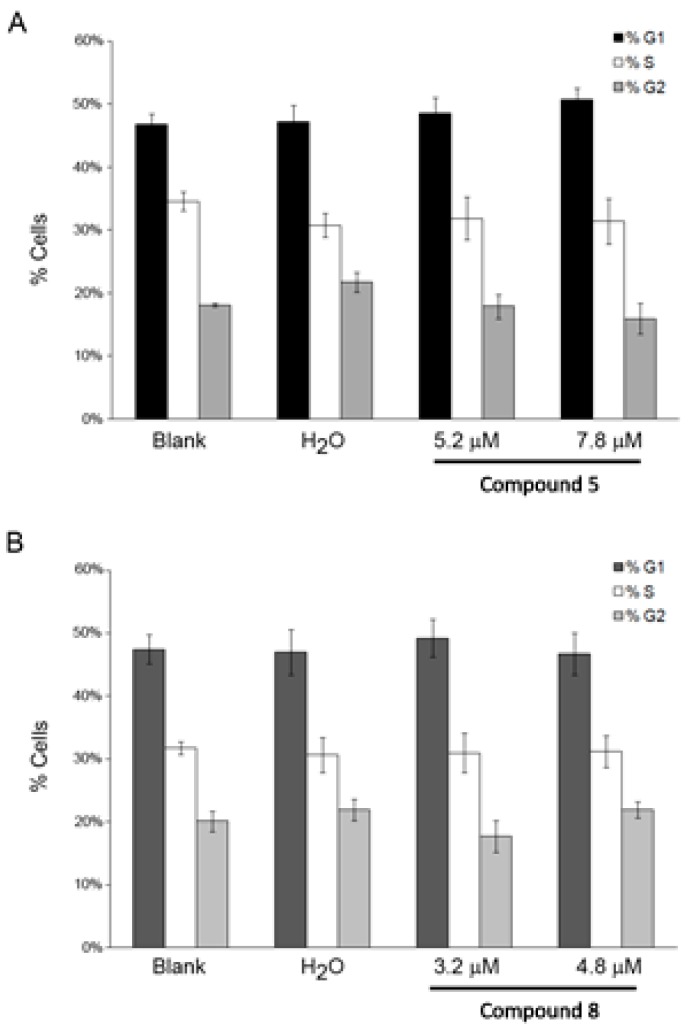
NCI-H460 cell cycle profile 48 h following treatment with compounds **5** (**A**) and **8** (**B**), analyzed by flow cytometry. Cells were treated with the GI_50_ (5.2 μM) and 1.5× GI_50_ (7.8 μM) of compound **5** and with the GI_50_ (3.2 μM) and 1.5× GI_50_ (4.8 μM) of compound **8**. Cells were also treated with the corresponding highest concentration of the vehicle (solvent) of the compounds (H_2_O). Results represent the mean ± SEM of at least three independent experiments.

To determine whether the effect of compounds on cell proliferation was related to cell cycle control, we analyzed the effects on cell cycle in NCI-H460 cells at 48 h after drug treatment by flow cytometry. As shown in Figure 2, the percentages of cells in each cell cycle phase were similar to untreated cells, indicating that the compounds did not affect cell cycle profile.

**Figure 3:**
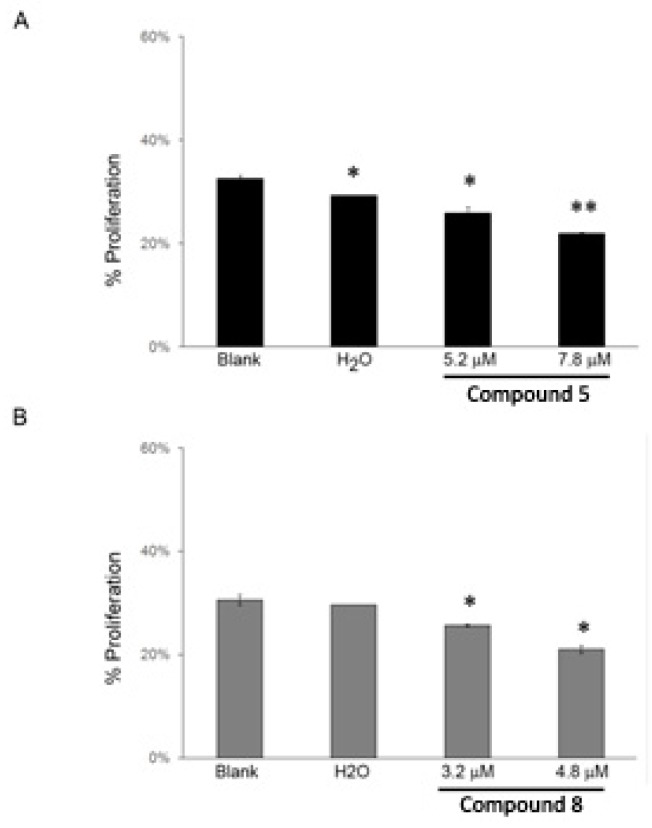
NCI-H460 cellular proliferation following 48 h treatment with compounds **5** (**A**) and **8** (**B**), analyzed with the BrdU incorporation assay. Cells were treated with the GI_50_ (5.2 μM) and 1.5× GI_50_ (7.8 μM) of compound **5** and with the GI_50_ (3.2 μM) and 1.5× GI_50_ (4.8 μM) of compound **8**. Cells were also treated with the corresponding highest concentration of vehicle (solvent) of the compounds (H_2_O). Results represent the mean ± SEM of three independent experiments. * *p* ≤ 0.001, ** *p* ≤ 0.05 between Blank vs. treatment.

Detection and quantification of cells actively synthesizing DNA in the S-phase of cell cycle progression is important in defining the cellular responses to drug treatments, assessing cell health, and determining genotoxicity. Thus, we have performed the BrdU incorporation assay [15,16] in NCI-H460 treated cells. A statistically significant decrease in cellular proliferation was observed after treatment with both compounds (Figure 3). Particularly, for compound **5** the percentage of BrdU-incorporating cells decreased from 32% (in untreated cells) to 25% and 22% (with the GI_50_ and 1.5× GI_50_ treatments, respectively), and for compound **8** the percentage of BrdU-incorporating cells decreased from 31% (in untreated cells) to 26% and 21% (with the GI_50_ and 1.5× GI_50_ treatments, respectively), indicating a dose-dependent decrease of cell proliferation after compounds exposure.

### 2.6. Effect of Compounds ***5*** and ***8*** on HSP90 Client Proteins

The effect of compounds in cellular apoptosis/proliferation led us to the analysis of HSP90 client proteins involved in those mechanisms.

**Figure 4:**
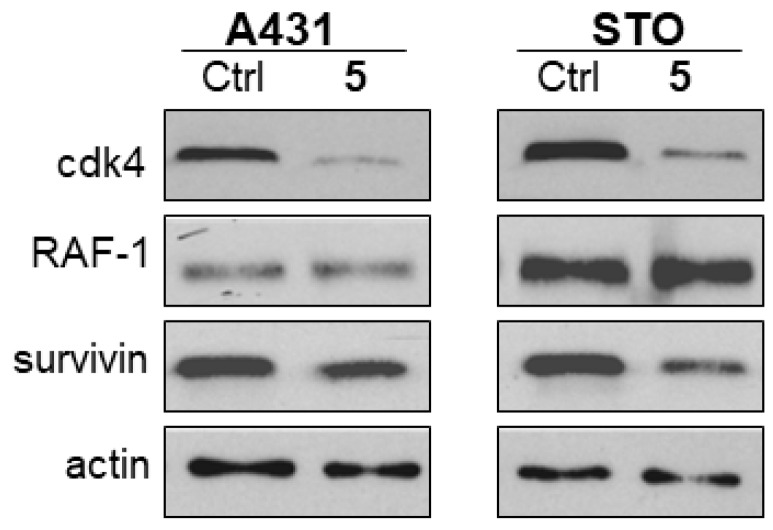
Analysis of HSP90 client protein levels in squamous-cell carcinoma (A431) and peritoneal mesothelioma (STO) cells after 24 h of treatment with compound **5** (5.4 μM in A431 cells; 2.7 μM in STO cells). Total cellular extracts were obtained 24 h after cell treatment. Actin is shown as a control for protein loading.

**Figure 5:**
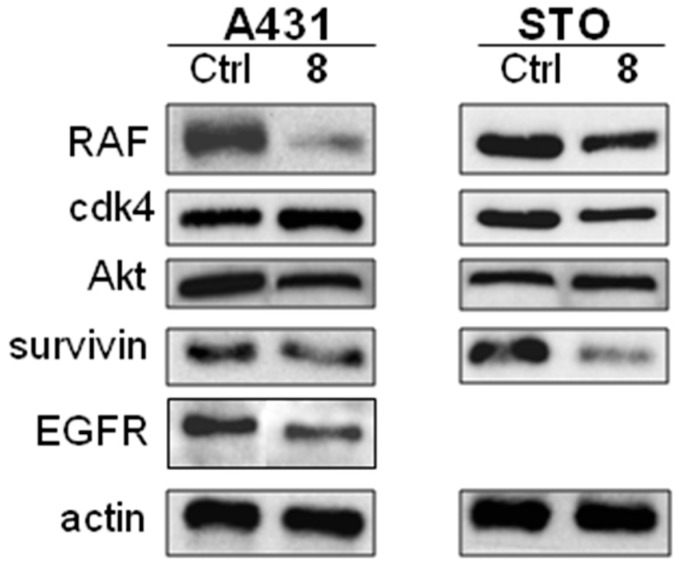
Analysis of HSP90 client protein levels in A431 and STO cells after 24 h of treatment with compound **8** (2.6 μM in A431 cells; 2.6 μM in STO cells). Total cellular extracts were obtained 24 h after cell treatment Actin is shown as a control for protein loading.

The most effective anti-proliferative agents, i.e., compounds **5** and **8**, were investigated for their ability to downregulate selected proteins known as “clients” of HSP90. As expected on the basis of the putative mechanism of action, the tested compounds induced a partial downregulation with a different pattern of inhibition. Specifically, compound **5** induced an almost complete downregulation of CDK4 and a partial downregulation of survivin in STO and A431 cells (Figure 4). Compound **8** still caused degradation of survivin in STO cells, but the effect was less marked in A431 cells. In the latter cell line, the most evident effects were a partial downregulation of Akt and EGFR and a strong downregulation of RAF (Figure 5). The different pattern of HSP90 client protein downregulation after treatment with compounds **5** or **8** (in both cell lines) is most likely due to their different physico-chemical features, which may most likely influence the interactions at a cellular level and, as a consequence, the activity of the compounds and the effect on client protein levels. In addition, the differences observed in the effect of the compounds between the two cell lines may be due to different basal levels of expression of these proteins between the two cell lines. Nevertheless, the observed modulations are consistent with an effect mediated by interaction of the selected compounds with HSP90.

### 2.7. Effect of Compound ***5*** in the Growth of a Tumor Xenograft Mouse Model

**Figure 6:**
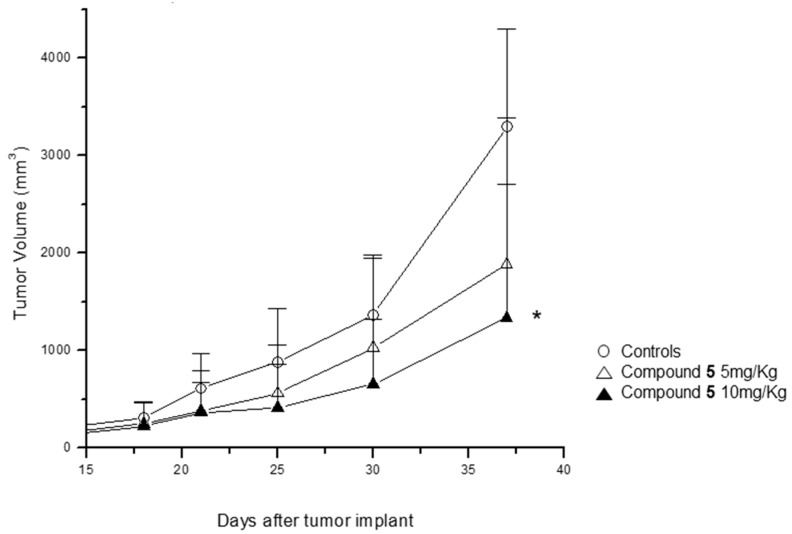
Antitumor activity of compound **5** in A431 squamous cell carcinoma xenografts in nude mice. Compound **5** dissolved in physiologic saline was administered i.p. (5 mg/kg and 10 mg/kg) for five consecutive days/week. The treatment started when the tumors were just palpable and was repeated for three weeks.

On the basis of the cellular/biochemical profile described above (Table 1), the therapeutic effect of compound **5** was determined in a human epidermoid carcinoma xenografted mouse model. Two experimental groups were used: (i) animals administrated with saline solution and (ii) animals intraperitoneally administrated with newly synthesized compound. The daily administration of compound **5** (5 mg/kg) for three weeks resulted in a considerable inhibition of tumor growth when compared with the control group (Figure 6). The antitumor effect was dose-dependent, as the increase of the dose of compound **5** (to 10 mg/kg), accentuated this difference to statistically significant values. Interestingly, at both doses used in this study no evident manifestations of toxicity were detected (specifically, death and body weight loss).

## 3. Material and Methods

### 3.1. Chemistry

All reagents and solvents were reagent grade or were purified by standard methods before use. Melting points were determined in open capillaries. NMR spectra were recorded at 300 MHz. NMR spectra were recorded on Varian Mercury 300 MHz and Bruker AV600 spectrometers The accurate mass spectra were recorded using a Bruker Daltonic model ICR-FTMS APEX II. Solvents were routinely distilled prior to use; dry methylene chloride was obtained by distillation from phosphorus pentoxide and toluene from CaCl_2_. All reactions requiring anhydrous conditions were performed under a positive nitrogen flow, and all glassware was oven dried. Isolation and purification of the compounds were performed by flash column chromatography on silica gel 60 (230–400 mesh). Analytical thin-layer chromatography (TLC) was conducted on TLC plates (silica gel 60 F254, aluminium foil) and spots were visualized by UV light and⁄or by means of dyeing reagents. Compound **4** was prepared according to literature procedure [17].

Compound **7** was prepared according to literature procedure [18].

Compound **9** (1-azaxanthone) was purchased from Alfa Aesar (76057, Karlsruhe, Germany).

*2-Benzyl-5,10-dioxo-5,10-dihydro-benzo[g]isoquinolinium bromide* (**2a**): To a solution of benzo[g]isoquinoline-5,10-dione **1** (70 mg, 0.33 mmol) in anhydrous DMSO (4 mL), benzyl bromide (56 μL, 0.47 mmol) was added under nitrogen. The solution was stirred 48 h at room temperature. The mixture was cooled at 0 °C and washed with ethyl ether to remove benzyl bromide and DMSO. Compound **2a** (110 mg, 86% yield) was obtained after crystallization in ethyl ether; m.p. 227 °C, *R*_f_ = 0.35 (CH_2_Cl_2_:CH_3_OH 19.5:5), ^1^H-NMR (DMSO-*d*_6_, 300 MHz): δ: 10.01 (1H, s); 9.57 (1H, d, *J* = 6 Hz); 8.72 (1H, d, *J* = 6 Hz); 8.41–8.22 (2H, m); 8.12–7.98 (2H, m); 7.69–7.39 (5H, m); 6.11 (2H, s). ^13^C-NMR (DMSO-*d*_6_, 150 MHz): δ: 179.3, 179.2, 149.0, 145.0, 143.7, 135.6 (×2C), 134.0, 133.0, 132.7, 131.3, 129.6, 129.3 (×2C), 129.0 (×2C), 127.3, 127.1, 124.5, 64.0. Anal. Calcd. for: C_20_H_14_BrNO_2_: C, 63.18; H, 3.71; N, 3.68. Found: C, 63.25; H, 3.70; N, 3.67. HRMS (ES^+^) *m*/*z*: Calcd. for C_20_H_14_NO_2_ [M]^+^ 300.1025. Found: 300.1024.

*2-(2,4-Difluoro-benzyl)-5,10-dioxo-5,10-dihydro-benzo[g]isoquinolinium bromide* (**2b**): To a solution of benzo[g]isoquinoline-5,10-dione **1** (15 mg, 0.072 mmol) in anhydrous DMSO (1 mL), 2-bromomethyl-1,4-difluorobenzene (13 μL, 0.107 mmol) was added under nitrogen. The solution was stirred 24 h at room temperature. The mixture was cooled at 0 °C and washed with ethyl ether to remove benzyl bromide and DMSO. Compound **2b** (60%) was obtained after cristallization in CH_3_OH/ethyl ether m.p. 210 °C dec, *R*_f_ = 0.32 (CH_2_Cl_2_:CH_3_OH 19.5:5) ^1^H-NMR (DMSO-*d*_6_, 300 MHz): δ: 9.99 (1H, s); 9.49 (1H, d, *J* = 6.0 Hz); 8.70 (1H, d, *J* = 6.0 Hz); 8.38–8.221 (2H, m); 8.12–8.00 (2H, m); 7.89–7.25 (1H, m); 7.51–7.38 (1H, m); 7.33–7.19 (1H, m); 6.20 (2H, s). Anal. Calcd. for: C_20_H_12_BrF_2_NO_2_: C, 57.71; H, 2.91; N, 3.37. Found: C, 57.65; H, 2.92; N, 3.37. HRMS (ES^+^) *m*/*z*: Calcd. for C_20_H_12_F_2_NO_2_ [M]^+^ 336.0836. Found: 336.0834.

*2-(4-tert-Butyl-benzyl)-5,10-dioxo-5,10-dihydro-benzo[g]isoquinolinium bromide* (**2c**): To a solution of benzo[*g*]isoquinoline-5,10-dione **1** (22 mg, 0.105 mmol) in anhydrous DMSO (1.5 mL), 1-bromomethyl-4-*tert*-butyl-benzene (23 μL, 0.125 mmol) was added under nitrogen. The solution was stirred 24 h at room temperature. The mixture was cooled at 0 °C and washed with ethyl ether to remove benzyl bromide and DMSO. Compound **2c** (30 mg, 65% yield) was obtained after crystallization in CH_3_OH/ethyl ether. m.p. 238 °C dec, *R*_f_ = 0.29 (CH_2_Cl_2_:CH_3_OH 19.5:5) ^1^H-NMR (DMSO-*d*_6_, 300 MHz): δ: 10.11 (1H, s); 9.60 (1H, d, *J* = 6.1 Hz); 8.70 (1H, d, *J* = 6.1 Hz); 8.41–8.22 (2H, m); 8.12–7.98 (2H, m); 7.69–7.39 (4H, m); 6.08 (2H, s); 1.22 (9H, s). ^13^C-NMR (DMSO-*d*_6_, 75 MHz): δ: 179.4, 179.2, 149.3, 149.1, 145.2, 143.5, 135.6 (× 2C), 134.1, 132.9, 132.5, 129.6, 129.2, 129.0 (×2C), 128.1 (×2C), 127.3, 127.0, 63.8, 34.3, 31.6 (×3C). HRMS (ES^+^) *m*/*z*: Calcd. for C_24_H_22_NO_2_ [M]^+^ 356.1651. Found: 356.1645. Anal. Calcd. for: C_24_H_22_BrNO_2_: C, 66.06; H, 5.08; N, 3.21. Found: C, 66.16; H, 5.09; N, 3.20. 

*2-Benzyl-10-oxo-10H-9-oxa-2-azonia-anthracene bromide* (**5**): To a solution of compound **4** (100 mg, 0.51 mmol) in anhydrous DMSO (2 mL), benzylbromide (90 μL, 0.75 mmol) was added under nitrogen. The solution was stirred overnight at room temperature. The mixture was cooled at 0 °C and washed with hexane and ethyl ether to remove benzyl bromide and DMSO. Compound **5** (161 mg, 86% yield) was obtained after crystallization in CH_3_OH/ethyl ether; m.p. = 231 °C, *R*_f_ = 0.43 (CH_2_Cl_2_:CH_3_OH 9:1). ^1^H-NMR (DMSO-*d*_6_, 300 MHz): δ: 10.22 (1H, s); 9.13 (1H, d, *J* = 5.5 Hz); 8.70 (1H, d, *J* = 5.5 Hz); 8.25 (1H, dd, *J* = 8.0 Hz); 8.05 (1H, dd, *J* = 8, 8.3 Hz); 7.85 (1H, d, *J* = 8.3 Hz); 7.71–7.58 (3H, m); 7.51–7.41 (3H, m); 6.06 (2H, s). ^13^C-NMR (DMSO-*d*_6_, 150 MHz): δ: 174.2, 156.1, 153.2, 140.2, 139.0, 138.0, 134.3, 131.6, 130.0, 129.6 (×2C), 129.4 (×2C), 126.7 (×2C), 124.7, 122.3, 119.1, 64.5. HRMS (ES^+^) *m*/*z*: Calcd. for C_19_H_14_NO_2_ [M]^+^ 288.1025. Found: 288.1024. Anal. Calcd. for: C_19_H_14_BrNO_2_: C, 61.97; H, 3.83; N, 3.80. Found: C, 62.03; H, 3.83; N, 3.79.

*2-Benzyl-5-oxo-5H-thiochromeno[2,3-c]pyridin-2-ium bromide* (**8**): To a solution of compound **7** (50 mg, 0.23 mmol) in anhydrous DMSO (1.5 mL), benzylbromide (40 μL, 0.34 mmol) was added under nitrogen. The solution was stirred overnight at room temperature. The mixture was cooled at 0 °C and washed with hexane and ethyl ether to remove benzyl bromide and DMSO. Compound **8** (50 mg, 55% yield) was obtained after crystallization in ethyl ether; m.p. 202 °C, *R*_f_ = 0.43(CH_2_Cl_2_:CH_3_OH 9:1). ^1^H-NMR (DMSO-*d*_6_, 300 MHz): δ: 10.21 (1H, s); 9.10 (1H, d, *J* = 5.3 Hz); 8.81 (1H, d, *J* = 5.5 Hz); 8.25 (1H, dd, *J* = 8.0 Hz); 8.05 (1H, dd, *J* = 8, 8.3 Hz); 7.85 (1H, d, *J* = 8.3 Hz); 7.71–7.58 (3H, m); 7.51–7.41 (3H, m); 6.06 (2H, s).^13^C-NMR (DMSO-*d*_6_, 150 MHz): δ: 176.4, 146.1, 139.9, 137.9, 137.0, 135.6, 134.5, 133.7, 129.6, 129.3 (× 2C), 129.2 (× 2C), 129.1, 128.6, 128.3, 127.5, 126.4, 63.9. HRMS (ES^+^) *m*/*z*: Calcd. for C_19_H_14_NOS [M]^+^ 304.0786. Found: 304.0799. Anal. Calcd. for: C_19_H_14_BrNOS: C, 59.38; H, 3.67; N, 3.64. Found: C, 59.31; H, 3.66; N, 3.65.

*1-Benzyl-10-oxo-10H-9-oxa-1-azonia-anthracene bromide* (**10**): To a solution of compound **9** (40 mg, 0.20 mmol) in anhydrous DMSO (1.5 mL), benzylbromide (30 μL, 0.25. mmol) was added under nitrogen. The solution was stirred overnight at room temperature. The mixture was cooled at 0 °C and washed with hexane and ethyl ether to remove benzyl bromide and DMSO. Compound **10** (25 mg, 34% yield) was obtained after crystallization in ethyl ether. ^1^H-NMR (DMSO-*d*_6_, 300 MHz): δ: 9.53 (1H, d, *J* = 4.5 Hz); 9.26 (1H, d, *J* = 6.5 Hz); 8.27 (1H, d, *J* = 7.1 Hz); 8.21–7.99 (3H, m); 7.82–7.60 (3H, m); 7.11–7.38 (3H, m); 6.12 (2H, s). m.p. 240 °C, *R*_f_ = 0.47 (CH_2_Cl_2_:CH_3_OH 9:1). Anal. Calcd. for: C_19_H_14_BrNO_2_: C, 61.97; H, 3.83; N, 3.80. Found: C, 61.92; H, 3.82; N, 3.81. HRMS (ES^+^) *m*/*z*: Calcd. for C_19_H_14_NO_2_ [M]^+^ 288.1025. Found: 288.1018.

### 3.2. Cell Lines and Culture Conditions

For the cytotoxic activity studies and determination of GI_50_ concentrations in three different tumor cell lines, the cell culture conditions are described in Section 3.5. For the studies on the effect on % viable cells, cell cycle, cellular proliferation, and apoptosis, culture conditions are described in Section 3.6.1. For the remaining studies, the epithelial carcinoma cell line A431 and the non-small cell lung carcinoma cell line NCI-H460 were routinely grown in RPMI 1640 (Lonza, Vierviers, Belgium), supplemented with 10% (*v*/*v*) heat-inactivated fetal bovine serum (FBS, GIBCO, Invitrogen, Paisley, UK). The human peritoneal mesothelioma cell line STO was kindly provided by Dr. N. Zaffaroni (IRCCS Istituto Nazionale Tumori, Milan, Italy) and grown in 50/50 DMEM-Ham’s F12 medium (Lonza, Vierviers, Belgium), supplemented with 10% (*v*/*v*) heat-inactivated foetal bovine serum and 2 mM l-glutamine (l-Gln). All cell lines were maintained at 37 °C in a 5%/95% CO_2_/air atmosphere. All the compounds tested were freshly prepared by dissolving powder in dimethylsulfoxide (DMSO) (BDH Prolabo, Milan, Italy) and following dilution in culture medium. Cells were incubated with drugs for 48 h or 72 h (unless otherwise stated) at 37 °C in culture medium supplemented with 10% (*v*/*v*) heat-inactivated FBS; drug concentrations used for each assay and duration of treatments are reported in Legends to Figures.

### 3.3. Cell-Counting Assay and Determination of IC_50_ Concentrations

Cellular sensitivity to drugs was evaluated by a cell-counting assay after 72-h drug exposure. Cells in the logarithmic phase of growth were seeded in duplicate into 6-well plates. Twenty-four hours after seeding, the drug was added to the medium. Cells were harvested 72 h after drug exposure and counted with a cell counter. IC_50_ is defined as the drug concentration causing a 50% reduction of cell number compared with that of untreated control.

### 3.4. HSP90 Binding Activity

GM-FITC Geldanamycin-fluorescein-5-isothiocyanate (working concentration: 1–400 nM), supplied by Invivogen (06C23-MT, San Diego, CA, USA), was previously dissolved in DMSO to obtain 10 mM stock solutions and kept at −20 °C until use. HSP90, purchased from (Stressgen cat. No. SPP-776, Victoria, BC, Canada), was previously dissolved in assay buffer (HFB) to form 2.2 mM stock solutions and kept at −80 °C until use. The compounds were previously dissolved in DMSO to obtain stock solutions and kept at −20 °C. The day of experiment, the compounds were prepared by serial dilutions in assay buffer (HFB) containing 20 mM HEPES (K) pH 7.3, 50 mM KCl, 5 mM MgCl_2_, 20 mM Na_2_MoO_4_ and 0.01% NP40. Before each use, 0.1 mg/mL Bovine Gamma globulin and 2 mM DTT were freshly added. Fluorescence Polarization (FP) was performed in Opti-Plate™-96F well plates (Perkin Elmer, Zaventem, Belgium) using a plate reader (Wallac Envision 2101 multilabel reader, Perkin Elmer, Zaventem, Belgium). To evaluate the binding affinity of the molecules, 50 mL of the GM-FITC solution (5 nM) were added to 30 nM of HSP90 in the presence of 5 μL of the test compounds at increasing concentrations. The plate was mixed on a shaker at 4 °C for 4 h, and the FP values in mP (millipolarization units) were recorded. The IC_50_ values were calculated as the inhibitor concentration where 50% of the tracer is displaced; each data point is the result of the average of triplicate wells, and was determined from a plot using nonlinear least-squares analysis. Curve fitting was performed using Prism GraphPad software program (GraphPad software, Inc., San Diego, CA, USA).

### 3.5. Analysis of Cell Growth Inhibition with the Sulforhodamine B Assay and Determination of GI_50_ Concentrations

The following human tumor cell lines were used and maintained in RPMI-1640 medium with UltraGlutamine I and 25 mM Hepes (Lonza, Basel, Switzerland), supplemented with 5% FBS (Biowest): NCI-H460 (non-small cell lung adenocarcinoma), AGS (gastric adenocarcinoma) and MCF-7 (breast adenocarcinoma). The HFF-1 cell line (human foreskin fibroblasts) was cultured in DMEM High Glucose with l-Glutamine supplemented with 15% FBS. All cell lines were routinely maintained at 37 °C in a humidified incubator with 5% CO_2_. Cell number and viability were determined with the trypan blue exclusion assay. All the experiments were performed with exponentially growing cells presenting more than 90% cell viability. 

The growth inhibitory effect of the compounds was assessed using the Sulforhodamine B (SRB) assay as previously described [19]. Briefly, cells were plated into 96-well plates (5000 cells/well for MCF-7 and NCI-H460 cells and 7500 cells/well for AGS cells) and incubated for 24 h to adhere. Serial dilutions of the different compounds (ranging from 0 to 150 μM or from 0 to 50 μM) were then added to the cells. Cells were further incubated for 48 h. Following this period (or at the time the compound is added, T_0_), plates were fixed with 10% ice-cold trichloroacetic acid. Following staining with 0.4% SRB, plates were washed with 1% acetic acid and the bound dye solubilized with 10 mM Tris-Base. The corresponding absorbance was measured at 510 nm in a microplate reader (Biotek Instruments Inc., Synergy XS, Winooski, VT, USA), allowing to determine the GI_50_ concentrations (corresponding to the concentration which results in 50% cell growth inhibition) for each of the studied compounds. For the HFF-1 cell line, cells (15,000 cells/well) were plated into 96-well plates, allowed to adhere for 24 h and treated with the GI_50_ concentration of each compound for seven days. After this period, the SRB assay was carried out as described above. The effect of the maximum concentration of vehicle (solvent) of the compounds (H_2_O: 0.31% for compound **8** and 0.19% for compound **13**) was also analyzed in each assay.

### 3.6. Effect of Compounds ***5*** and ***8*** on Cell Cycle Profile, Cellular Proliferation and Apoptosis in NCI-H460 Cells

#### 3.6.1. Drug Treatments

NCI-H460 cells were plated in 6-well plates (1 × 10^5^ cells/well) in RPMI-1640 medium with UltraGlutamine I and 25 mM Hepes (Lonza) supplemented with 10% FBS (PAA) at 37 °C in a humidified incubator with 5% CO_2_ for 24 h. Cells were then treated for 48 h with the GI_50_ and 1.5× GI_50_ concentrations of compounds **5** and **8**. These concentrations corresponded respectively to 5.2 μM and 7.8 μM for compound **5** and to 3.2 μM and 4.8 μM for compound **8**. Cells were also incubated with medium only (Blank) or with the vehicle (solvent, H_2_O), corresponding to the highest concentration used when testing the compounds. Following treatment with the compounds, cells were further processed as follows. 

#### 3.6.2. Determination of % Viable Cells

Cell number and % viable cells was determined with the Trypan blue exclusion assay.

#### 3.6.3. Analysis of Cell Cycle Profile 

Following 48 h treatment, cells were fixed in ice-cold 70% ethanol and stored at 4 °C at least overnight. Cell pellets were incubated in 5 μg/mL propidium iodide and 100 μg/mL RNase A (in PBS) for 30 min on ice. Analysis of cellular DNA content was carried out using a FACSCalibur flow cytometer (BD Biosciences, Erembodegem, Belgium). The FlowJo 7.6.5 software (Tree Star, Inc., Ashland, OR, USA) was used to determine the % of cells in the different phases of the cell cycle (G1, S and G2/M phases of the cell cycle) after exclusion of cell debris and aggregates and analyzing at least 15,000 events [20].

#### 3.6.4. Analysis of Cellular Proliferation

Following 47 h treatment, cells were further incubated with 10 μM bromodeoxyuridine (BrdU; Sigma) for 1 h. Cells were then trypsinized and fixed in 4% paraformaldehyde (in PBS) for 30 min. Cytopsins were prepared and a 20 min denaturation step was carried out by incubating slides in 2 M HCl. Following incubation with mouse anti-BrdU primary antibody (1/10, Dako) for 1 h at room temperature (RT), cells were further incubated with anti-mouse-Ig-FITC (1/100, Dako) for 30 min, at RT [21]. Slides were then mounted with Vectashield mounting medium with DAPI (Vector Laboratories). The percentage of BrdU incorporating cells was determined by counting a minimum of 500 cells per slide (except for one experiment in which due to technical difficulties less cells were counted when testing the compound **8**) using a fluorescence microscope (DM2000; Leica, Wetzlar, Germany ) [22]. 

#### 3.6.5. Analysis of Apoptosis

Following 48 h treatment, the levels of apoptosis were assessed using the “Human AnnexinV-FITC/PI apoptosis” kit (Bender MedSystems, Wien, Austria) as previously described [23]. Briefly, cell pellets were resuspended in binding buffer and further incubated with Annexin V-FITC and with propidium iodide. Samples were analyzed in a FACSCalibur flow cytometer (BD Biosciences, Erembodegem, Belgium), plotting at least 15,000 events and using the FlowJo 7.6.5 software (Tree Star, Inc., Ashland, OR, USA) [24].

### 3.7. Effect of Compounds ***5*** and ***8*** on Protein Expression Analyzed by Western Blot

Total cell lysates were prepared as described previously [25]. Primary antibodies used in this study are: anti-Raf-1 and anti-CDK4 (Santa Cruz Biotechnology Inc., Santa Cruz, CA, USA), anti-Survivin (Abcam, Cambridge, UK), anti-Akt (Transduction Laboratories, Lexington, KY, USA), and anti-Actin Sigma Chemical Co., St. Louis, MO, USA).

### 3.8. Analysis of the Effect of Compound ***5*** on the Tumor Growth of Xenografted Mouse Models

Experiments were carried out using female athymic Swiss nude mice, 8–10 weeks old (Charles River, Calco, Italy). Mice were maintained in laminar flow rooms keeping temperature and humidity constant. Mice had free access to food and water. Experiments were approved by the Ethics Committee for Animal Experimentation of the Istituto Nazionale Tumori of Milan according to institutional guidelines. Epidermoid carcinoma cells, A-431 suspended in culture media were subcutaneously injected on the back of mice. When tumors were just palpable, the mice were randomly divided into two groups: control and compound-treated. Each group was constituted by 8 mice. Saline (0.9% NaCl) or compound solution (5 or 10 mg/kg, respectively) was i.p. administered daily during 3 weeks, to both groups. Tumor volume was assessed using calipers and determined according to the formula (d)^2^xD/2.

### 3.9. Statistical Analysis

For the results regarding determination of GI_50_ concentrations, % viable cells, cellular proliferation and apoptosis, data is presented as mean ± standard error (SEM) from at least three independent experiments. For those experiments, statistical analysis was carried out using an unpaired student’s *t*-test. 

## 4. Conclusions

The present study describes the screening of a series of compounds with new chromenopyridinone and thiochromenopyridinone scaffolds as potential HSP90 inhibitors. Our results indicated that compounds **5** and **8** with a chromenopyridinone and thiochromenopyridinone skeleton, respectively, are promising regarding in vitro tumor cell growth inhibitory activity. Treatment with these compounds decreased the proliferation of tumor cells and compound **8** induced apoptosis. In addition, the compounds were able to downregulate selected proteins known as “clients” of HSP90. Specifically, compound **5** induced an almost complete downregulation of CDK4 and a partial downregulation of survivin in STO and A431 cells, whereas compound **8** produced degradation of survivin in STO cells and a significant downregulation of RAF in A431 cells. The observed modulations are consistent with an effect mediated by interaction of the selected compounds with HSP90. In addition, treatment of xenografted mice with compound **5** resulted in a considerable dose-dependent inhibition of tumor growth. Work is in progress to investigate structural changes of the heterocyclic scaffold, as well as of the *N*-substituent, and to improve the activity of the compounds. In addition, future work will confirm if these compounds inhibit the ATPase activity of purified HSP90 in vitro.

## Figures and Tables

**Table 1:** Binding affinity (μM) to heat shock protein 90 (HSP90) protein and cell growth inhibitory activity (μM) of the compounds.

	Binding Affinity (μM)	Cell Growth Inhibitory Activity (μM)		
Compound	Binding HSP90 (FP) IC_50_	NCI-H460 IC_50_ 72 h	STO IC_50_ 72 h	A431 IC_50_ 72 h
**2a**	n.a.	0.7 ± 0.01	n.d.	0.2 ± 0.01
**2b**	n.a.	3.2 ± 0.5	n.d.	3.1 ± 0.5
**2c**	n.a.	n.d.	n.d.	0.46 ± 0.8
**4**	>100	>50	>50	>50
**5**	n.a.	4.8 ± 0.1	2.7 ± 0.8	1.36 ± 0.05
**7**	2.47 ± 0.09	>50	46.9	46.9
**8**	3.7 ± 0.03	n.d.	0.26 ± 0.08	0.26 ± 0.40
**10**	>100	>50	>50	>50
**17-AAG**	1.095 ±0.05	0.010 ± 0.002	-	0.069 ± 0.005

n.a. = non-available results since the compounds were autofluorescent; n.d. = not determined; 17-AAG = tanespimycin, 17-*N*-allylamino-17-demethoxygeldanamycin.

**Table 2:** GI_50_ values of compounds **5** and **8** following 48 h treatment of three human tumor cell lines.

Compound	GI_50_ (μM)		
	MCF7	NCI-H460	AGS
**5**	5.4 ± 0.6	5.2 ± 1.2	6.9 ± 1.2
**8**	3.2 ± 0.1	3.2 ± 0.1	3.1 ± 0.3

Data refers to mean ± SEM of at least three independent experiments. The maximum vehicle concentration used (H_2_O or DMSO; 0.25%) was also analyzed as control treatments and presented no effect on cell growth (data not shown).

**Table 3:** Levels of apoptosis in non-small cell lung cancer (NCI-H460) cells following 48 h treatment with compounds **5** (A) and **8** (B).

(A)	Treatments	% Apoptosis	(B)	Treatments	% Apoptosis
Blank		7.0 ± 0.6	Blank		5.2 ± 0.9
H_2_O		7.6 ± 1.2	H_2_O		6.4 ± 1.8
Compound 5	5.2 μM	7.2 ± 1.4	Compound 8	3.2 μM	6.7 ± 1.4
	7.8 μM	7.3 ± 0.9		4.8 μM	12.5 ± 1.7 **

Results are the mean ± SEM of at least 3 independent experiments; ** *p ≤* 0.05 between Blank vs. treatment; % Apoptosis refers to the sum of early and late apoptosis.
